# Mechanism of Inflammation in Age-Related Macular Degeneration

**DOI:** 10.1155/2012/546786

**Published:** 2012-11-07

**Authors:** Francesco Parmeggiani, Mario R. Romano, Ciro Costagliola, Francesco Semeraro, Carlo Incorvaia, Sergio D'Angelo, Paolo Perri, Paolo De Palma, Katia De Nadai, Adolfo Sebastiani

**Affiliations:** ^1^Department of Ophthalmology, University of Ferrara, Ferrara, Italy; ^2^Department of Health Sciences, University of Molise, Campobasso, Italy; ^3^Department of Ophthalmology, Istituto Clinico Humanitas, Milan, Italy; ^4^Department of Ophthalmology, University of Brescia, Brescia, Italy; ^5^Center for Retinitis Pigmentosa of Veneto Region, ULSS 15 Alta Padovana, Camposampiero, Italy

## Abstract

Age-related macular degeneration (AMD) is a multifactorial disease that represents the most common cause of irreversible visual impairment among people over the age of 50 in Europe, the United States, and Australia, accounting for up to 50% of all cases of central blindness. Risk factors of AMD are heterogeneous, mainly including increasing age and different genetic predispositions, together with several environmental/epigenetic factors, that is, cigarette smoking, dietary habits, and phototoxic exposure. In the aging retina, free radicals and oxidized lipoproteins are considered to be major causes of tissue stress resulting in local triggers for parainflammation, a chronic status which contributes to initiation and/or progression of many human neurodegenerative diseases such as AMD. Experimental and clinical evidences strongly indicate the pathogenetic role of immunologic processes in AMD occurrence, consisting of production of inflammatory related molecules, recruitment of macrophages, complement activation, microglial activation and accumulation within those structures that compose an essential area of the retina known as macula lutea. This paper reviews some attractive aspects of the literature about the mechanisms of inflammation in AMD, especially focusing on those findings or arguments more directly translatable to improve the clinical management of patients with AMD and to prevent the severe vision loss caused by this disease.

## 1. Introduction

**Figure 1:**
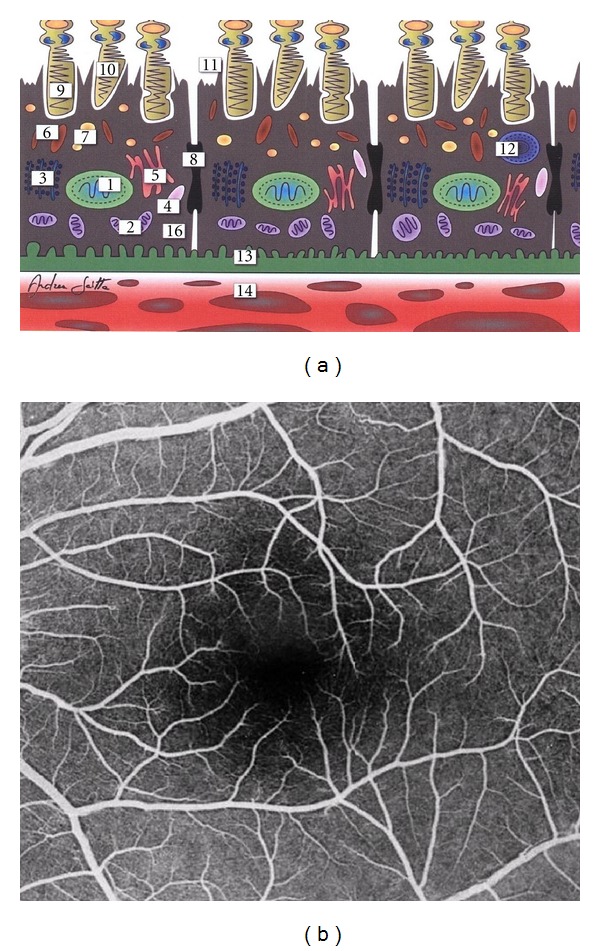
Normal human macula. (a) Schematic cross-sectional illustration of the macular outer segment and, in particular, the cells of the retinal pigment epithelium ((1) nucleus, (2) mitochondria, (3) ribosomes, (4) lysosomes, (5) Golgi apparatus, (6) melanosomes, (7) lipofuscin granules, (8) zonula occludens, (9) photoreceptor (cone), (10) outer segment of cones, (11) phagocytosis of photoreceptorial discs, (12) phagosome, (13) Bruch's membrane, and (14) choriocapillaris). (b) Fluorescein angiography of the macula with its foveal avascular zone (extracted and modified from [4]).

Age-related macular degeneration (AMD) is a common disease of the central area in the ocular posterior segment, known as the *macula lutea*. This retinal area is essential for the vision of fine details and the image resolution, capturing the greatest focus of the external light stimuli. In the human macula, there are several recognizable main structures: the neuroretina (NR), composed by the inner neurosensory layer and outer photoreceptor cell layer with the underlying retinal pigment epithelium (RPE); this latter structure is separated from the choriocapillaris (CC) by the Bruch's membrane (BM), a modified basement stratum. The retina contains two types of photoreceptors, rods and cones. The rods are more numerous (about 120 million) and are more sensitive than the cones, being responsible for vision at low light levels (scotopic vision). They are not sensitive to colors and characterized by low spatial acuity. Conversely, the 6 to 7 million cones are active at higher light levels (photopic vision), are capable of color vision, and are responsible for high spatial acuity. The center of the macula, called *fovea centralis*, is an avascular zone exclusively populated by cones (Figures 1(a) and 1(b) [4]). AMD is due to multifaceted degenerative disorders involving the NR-RPE-BM-CC complex at the level of macular region [1–3].

AMD represents the main cause of legal blindness or low vision in those developed countries with the longest life expectance, especially affecting the elderly people of European descent [3]. In North America, Europe, and Australia, AMD accounts for up to 50% of all cases of central blindness [5], approximately reaching a prevalence of 3% among the general adult population [6]. In the United States, about 15% of people older than 80 years of age were estimated to have AMD on 2000, and this number is expected to rise in the next years reaching more than 2.95 million people with AMD in 2020 [7].

Numerous and heterogeneous pathological processes are likely to predispose an individual to AMD, which is considered an extremely complex, multifactorial disease. Aging represents its primary determinant, while environmental factors such as cigarette smoking [8, 9], dietary habits [10–12], and phototoxic exposure [13–15] contribute to significantly increase the risk of AMD occurrence, together with several gene polymorphisms [16–22]. In a population-based twin study including both concordant/discordant and monozygotic/dizygotic sibling pairs, Seddon and coworkers have evaluated the relative contribution of heredity and environment to AMD etiology, concluding that heritability estimates for AMD are remarkable and range from 46% to 71% [23]. More recently, the same research group has indicated that individual genotypic susceptibility interacts with behavioral and nutritional factors in the etiology of AMD by means of various epigenetic mechanisms [24], further supporting the importance of the epigenetics into AMD investigations [25, 26]. These data, along with findings of genome-wide association studies, emphasize the presence of an important rationale to practice the search for AMD-related gene variants [27, 28], despite the unavoidable efforts required to plan genetic analyses of a complex disease with late onset. In particular, remarkable correlations were documented between common or rare immunological/inflammatory gene polymorphisms and AMD, unequivocally indicating the involvement of inflammation and immune-mediated processes (complement activation) in the pathogenesis of this disease [21, 29–32]. Thus, although AMD is not considered a classic inflammatory disease, immunocompetent cells, such as macrophages and lymphocytes, are present in the chorioretinal tissues affected by AMD [33, 34]. Moreover, peculiar signs of abnormalities/dysregulation of innate immune system are observed in eyes with AMD principally at the level of the complement pathway, including complement components C3a and C5a, C5 and C5b-9 terminal complement complex, complement regulators or inhibitors, that is, complement factor H (CFH), vitronectin and clusterin, complement receptor 1 (CR1, also called CD35), membrane cofactor protein (MCP, also called CD46), and decay accelerating factor (DAF, also called CD55), but also at the level of C-reactive protein (CRP) [18, 35–41]. In particular, activation products C3a, C5a, and C5b-9 are also systemically elevated in patients suffering from AMD [42–45]. In the course of AMD, several immunopathological phenomena occur within the NR-RPE-BM-CC complex of the macular area, especially due to the pathophysiologic effects of complement system, which have a main role in the parainflammation of the aging retina [46–52]. Herein, we briefly review the literature on the involvements of inflammation in AMD, highlight the possible environmental, genetic, and/or epigenetic interactions, and discuss those therapeutic approaches potentially able to modulate inflammatory pathways and more directly translatable to the management of AMD patients.

## 2. Parainflammation and Age-Related Macular Degeneration

Parainflammation is defined as a condition of tissue adaptive response to noxious stress or malfunction, and it has features which are considered as intermediate between normal/basal and inflammatory/acute states. Although the physiological purposes of normal parainflammation are to preserve tissues homeostasis and to restore their functionality, when a tissue is exposed to stress and/or malfunction for a prolonged period, it is implicated in both initiation and progression of many human age-related disorders, such as AMD [47, 53]. The risks of degenerative diseases, at least partially related to the pathophysiologic para-inflammatory response, are especially relevant in those tissues functionally dependent on nonproliferative cells and characterized by very high metabolism and other oxidative stress, such as the macular retina. In humans, the retina is a highly differentiated neuroectodermal tissue, in which an outer layer of photoreceptors, two layers of neuronal cells bodies, and two layers of synapses are present. The NR, together with RPE cells, forms the intraocular functional unit of the visual system. Like the components of central nervous system (brain and spinal cord) and several other tissues, retina also undergoes many pathophysiologic modifications with age. Because of cell and tissue damage/malfunction, mainly due to accumulative oxidative and metabolic changes in NR-RPE-BM-CC complex induced by reactive oxygen species (ROS), the vision sensitivity progressively declines during the aging process. In accordance with the “free radical theory of aging,” originally expressed by Harman in 1956, age-related degeneration is basically caused by an imbalance between ROS-induced tissue damages and repair/remodelling processes [54]. This concept seems to be extremely important for human AMD; in fact, its main risk factors include increased age, smoking, augmented body mass index, phototoxicity and inflammation [8–15, 55], and all these factors augment ROS generation [14, 56–62]. Moreover, exactly the innate immune system, which plays a key role in tissue repair/remodeling processes, is also the same one that is more interconnected with AMD susceptibility and etiopathogenesis starting, respectively, from genotypic [21, 29–32] and phenotypic [18, 35–45] points of view. Particularly, the outer photoreceptor/RPE/MB complex, that is, the site of onset of the elementary AMD lesions (drusen), is considered more prone to oxidative stress because of both its proximity with the highly variable choroidal hemodynamics and its continuous exposition to photooxidation due to light stimuli [13–15, 63, 64]. In fact, unregulated blood flow may increase the fluctuations of tissue oxygen concentration, leading to elevated ROS generation by the mitochondria [63, 64]. Likewise, photooxidation in photoreceptors is associated with complement activation [65], which can increase membrane attack complex formation, an important trigger of those apoptotic processes inducing nonlethal, retinal degeneration [66–68]. ROS augmentation can also trigger angiogenic signaling that has a crucial role in the occurrence of the more severe complication of AMD, that is, choroidal neovascularization (CNV) [69–72]. In other words, several factors, linked to AMD etiopathogenesis, lead to increased ROS generation and can mediate apoptosis and angiogenesis, which are more implicated in the atrophic and neovascular AMD forms, respectively [14, 46, 63–72]. Finally, the critical position of complement must be, once again, emphasized. In fact, dysregulation of complement pathways can lead to that autologous damage which, at the macular level, is manifested by the development of drusen. Starting from this rational (even if notional) point of view, the earliest hallmarks of AMD may act as foci of chronic inflammation [49, 52, 73].

**Figure 2:**
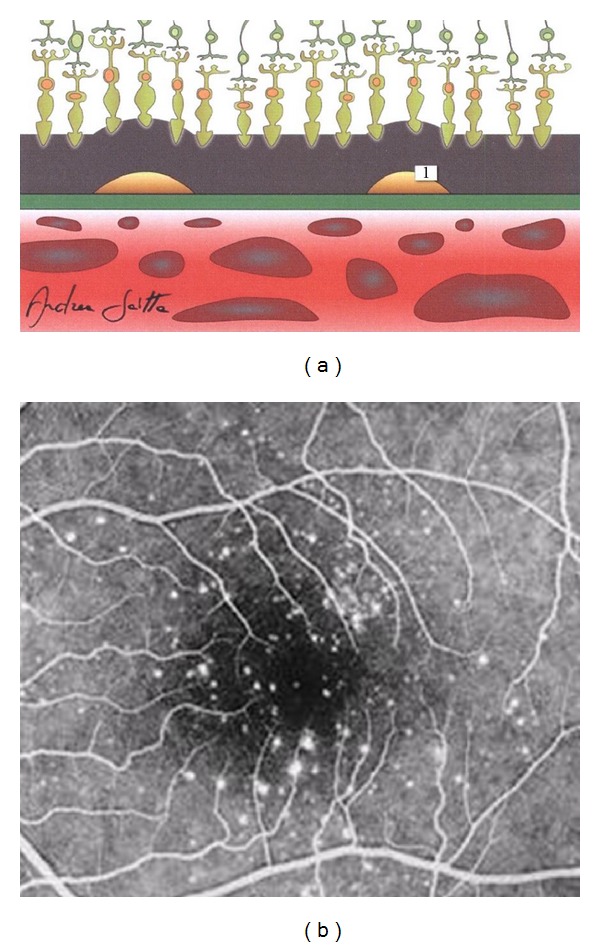
Early age-related macular degeneration. (a) Schematic cross-sectional illustration of the macula with an early stage of the disease ((1) drusen). (b) Fluorescein angiography of the macula affected by an early form of the disease (nonconfluent *hard* drusen); in this eye, the best best-correct visual acuity was 20/20 (Snellen equivalent) (extracted and modified from [4]).

**Figure 3:**
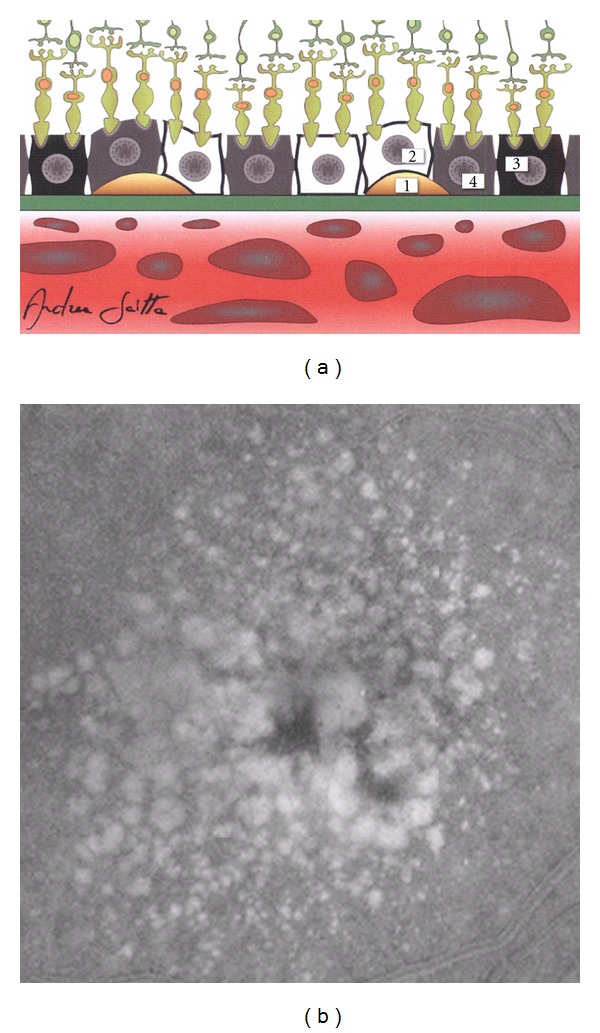
Intermediate age-related macular degeneration. (a) Schematic cross-sectional illustration of the macula with an intermediate stage of the disease ((1) drusen; (2) atrophy of a cell of the retinal pigment epithelium; (3) hypertrophy or hyperplasia of a cell of the retinal pigment epithelium; (4) a normal cell of the retinal pigment epithelium). (b) Fluorescein angiography of the macula affected by an intermediate form of the disease (confluent *soft* drusen and pigmentary irregularities); in this eye, the best best-correct visual acuity was 20/50 (Snellen equivalent) (extracted and modified from [4]).

**Figure 4:**
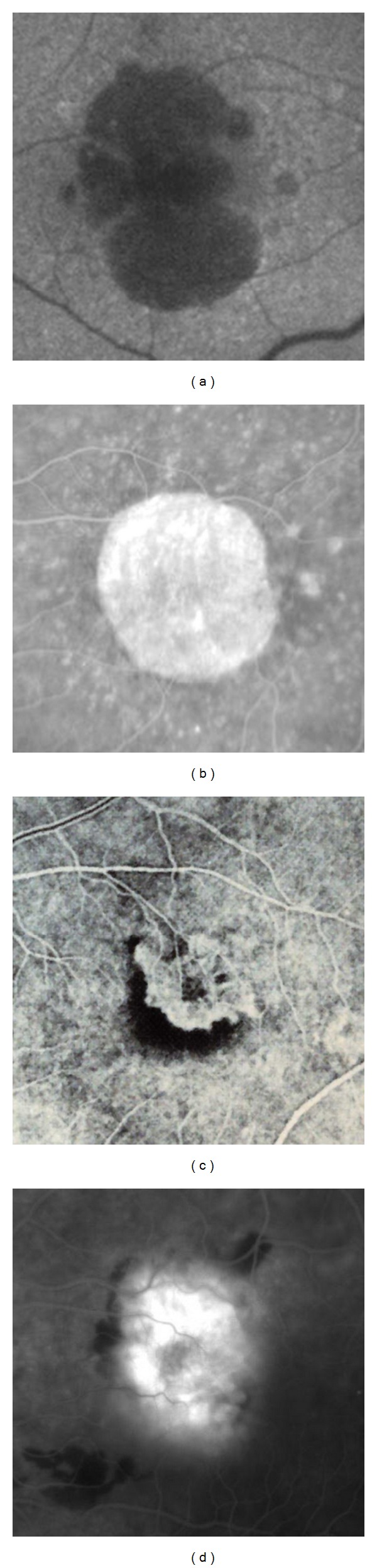
Advanced forms of age-related macular degeneration. (a) Autofluorescent retinography and (b) fluorescein angiography of two different cases of severe central geographic atrophy; in these eyes, the best-correct visual acuities were, respectively, 20/125 and 20/160 (Snellen equivalent). (c) Early and (d) late fluorescein angiograms of two different cases of subfoveal choroidal neovascularization; in both these eyes, the best-correct visual acuity was 20/200 (Snellen equivalent).

During the normal aging, in the NR, the number of neuronal and ganglion cells decreases, as also happens in the case of RPE cells which generally suffer the greatest losses in the macular and surrounding areas. Lipofuscin, the main aging-associated retinal end product, accumulates in the RPE cells with age, and its autofluorescent properties are routinely used in the clinical practice for the diagnostic imaging of various macular disorders. Another crucial age-related retinal change is the BM alteration, characterized by an increased thickness, accumulations of basal laminar deposits and/or drusen formation, and frequently accompanied by pigmentary irregularities due to RPE cell hypertrophy, hyperplasia, or atrophy. Usually, all these occurrences are more evident at the posterior pole in comparison with retinal periphery. In addition, both biochemical constitutions and biophysical properties of the BM modify with age, also influencing a further RPE cell dysfunction as well as noticeable CC disorders. Although the retina has been traditionally considered as an “immunologically privileged” tissue, at present it is known to have an endogenous immune system, actively coordinated by immunocompetent cells (microglia and dendritic cells), along with a rare population of perivascular macrophages; moreover, also RPE cells possess a variety of immunological functions. Retinal microglia and RPE cells, together with choroidal macrophages/dendritic cells, physiologically play an essential role in retinal homeostasis [47, 74–78]. In the aging retina, all these elements represent important factors in both dealing with the retinal malfunctions and restoring retinal homeostasis or rebalancing the homeostatic points. Several sight-threatening retinal diseases have a higher prevalence among the elderly persons, but the most common of these is AMD that can be diagnosed in its early (Figures 2(a) and 2(b) [4]) or intermediate (Figures 3(a) and 3(b) [4]) drusen/RPE-atrophy/pigmentary forms, as well as in its advanced forms, that is, geographic atrophy and neovascular AMD (Figures 4(a)–4(d)) [3]. Even if the clinical pattern of the above-mentioned types of AMD significantly differs, low-grade/subclinical degree of inflammation (parainflammation) is implicated in every AMD forms, reaching a high level when maculopathy is complicated by CNV development [71]. In neuroretinal structure, para-inflammatory modifications are characterized by the breakdown of blood-retinal barrier, microglial activation, and subretinal migration, whereas, in the choroid they become evident with an increased number of CD45^+^ CRIg^+^ macrophages, morphologic abnormalities of melanocytes, tissue's thickening, and fibrosis. At the retinal/choroidal interface, these AMD-related changes are particularly manifested by complement activation in RPE-BM cells and microglia accumulation in subretinal space [47, 48, 71]. Insightful knowledge on the mechanisms of retinal parainflammation, as well as of complement dysregulation, is fundamental to comprehensively understand the pathogenesis of AMD and to develop better curative therapeutic strategies for the different forms of this harmful disease.

## 3. Complement System and Age-Related Macular Degeneration

Complement system consists of over 40 proteins and regulators which are detectable in the blood circulation. It plays a key role in host defense against pathogens, adaptive immune responses, removal of the immune complexes and apoptotic cells [79]. In humans, three complement-mediated pathways complementarily act, and each of them is characterized by a specific trigger as follows:

- antibody-antigen complex for the classical pathway;
- binding to host cell or pathogen surface for the alternative pathway;
- polysaccharides on microbial surfaces for the lectin pathway.

Dysregulation and/or dysfunction of the complement pathways can result in various critical autologous damages, with consequent pathogenetic implications in a wide spectrum of diseases [52]. Both pathogenesis and progression of AMD represent complex events, in which complement system is directly or indirectly implicated. Pathobiologic studies have identified numerous complement proteins inside drusen (i.e., the elementary clinical lesions identifiable in the macula of AMD patients), and genetic analyses have discovered the existence of common or rare polymorphisms in several complement-related genes that significantly increase or reduce the risk for AMD late in life [49]. In fact, the phenotypic features of drusen (i.e., clinical pattern and time of onset) and the genotypic individual background for AMD seem to be mutually and closely intersected with each other, figuratively sharing, as lowest common denominator, the local dysregulation of the complement system in the NR-RPE-BM-CC complex due to acquired and/or inherited risk factors. In particular, the development of AMD-related drusen occurs between the basal surface of RPE and the BM, a single-stratified extracellular matrix in contact with CC (Figures 2(a) and 3(a) [4]) [80]. Since the mid 1990s, increasing experimental and clinical evidences clearly indicate that a lot of complement-related molecules, such as complement activators, complement components, and complement regulatory proteins, represent substantial constituents of the drusen [35, 36, 65, 66, 73, 81–93]. Starting from the beginning of the 2000s, the more and more exact identification of their compositional profile has been essential to create the basis for a new paradigm of AMD pathogenesis, in which macular and perimacular drusen should be considered as the earliest diagnosable byproducts of chronic local para-inflammatory phenomena at BM level. According to this model of AMD occurrence and progression, parainflammation of retinochoroidal tissues, accompanied by complement activation, immune-mediated processes, and bystander cell lysis, becomes the most crucial aspect of this neurodegenerative maculopathy [47–52, 73, 85, 94].

In the course of the past few years, a definitive support for the “immuno-inflammatory” model of AMD pathogenesis has been evidenced by the clinical-genetic findings of numerous studies, which revealed highly significant correlations between AMD and polymorphisms of genes encoding for several molecules directly involved in the activities of the complement alternative pathway. Of these genes/loci, the most studied ones are:

- complement factor H (CFH) [16–18, 95–99],
- complement component 3 (C3) [100–103],
- complement factor I (CFI) [104, 105],
- complement factor B (CFB) [19, 106, 107],
- complement component 2 (C2) [19, 106, 107],
- CFH-related genes (CFHR) type 1–5 [108–110].

Even if some of these relationships between these genes/loci and AMD are incompletely understood, their comprehensive consideration indicates, once again, that complement-related polymorphic alleles are able to increase (CFH, C3, CFI, and CFHR-2-4-5) or reduce (CFB, C2, and CFHR-1-3) AMD risk, representing a central key point on which the evidences of the high heritability of AMD are based [23, 106, 111]. Then again, genetic susceptibility to AMD is a very multifaceted issue that also includes several other immunological/inflammatory aspects, either just indirectly linked or not linked to complement system such as, for example:

- interactions between C-reactive protein (CRP) and Y402H variant of CFH gene (rs1061170), a very common single nucleotide polymorphism (SNP) located within the chromosome 1q32 region and unequivocally identified in association with AMD among multiple study populations—providing for the first time a logical basis by which to assess the disease's risk in over 50% of affected individuals [49, 50, 52, 112];
- potential synergisms between the above-mentioned SNPs in genes/loci encoding for factors or components of the alternative complement pathway and some noncomplement-related genes, located on the chromosome 10q26 region and extensively described as strongly implicated in AMD pathogenesis, that is, the rs10490924 SNP of the age-related maculopathy susceptibility 2 (ARMS2), and the rs11200638 SNP of the high-temperature requirement factor A of serine peptidase 1 (HTRA1) [21, 22, 26, 49, 111].

CRP is a biomarker of acute-phase inflammation. It plays an essential role in the innate immune response to tissue injury and/or infection. Because CRP induces complement activation via the alternative pathway, it is plausible that CRP may have a direct responsibility in AMD pathogenesis by causing macular damages via complement-mediated mechanisms, as also happens in the case of CFH [46, 50, 71, 113, 114]. In fact, although several facets of the CFH-CRP interaction are not yet well defined [115], several findings have indicated that in carriers of the polymorphic H402 variant of CFH gene a lower affinity for CRP exists in respect of the individuals with the Y402 protein [92, 116, 117]. Moreover, a more recent study has confirmed that native CRP-CFH interaction is evident at high plasma CRP concentrations (as happens during the acute-phase response, i.e., when the H402 protein inadequately binds to CRP) [118], and also a large meta-analysis has documented that serum levels of CRP >3 mg/L are related to a double AMD risk in comparison with CRP concentrations <1 mg/L [119]. Starting from these latter results, it is not surprising that homozygous CFH-Y402H polymorphic genotype, together with elevated serum/plasma CRP levels, leads to a very high risk of both AMD and its progression (with odds ratios of 19.3 and 6.8, resp.) [120], even if the CRP elevation is not related to any variant of the CRP gene and no polymorphism in this gene is directly associated with AMD [121, 122]. As well, at the levels of RPE and choroid of CFH-H402 homozygous carriers, a greater amount of CRP was detected in comparison with that found in Y402 homozygotes, but there was no significant difference in CFH protein concentrations among individuals with diverse Y402H genotypes. This lack of local CRP expression indicates that CRP is present in the posterior segment of the eye as a consequence of deposition through chronic low-grade local inflammation [91].

Based on early genome-wide linkage analyses, which have established that the 10q26 locus is closely associated with AMD [123–126], several clinical-genetic studies, specifically focused on this chromosomal region, discovered two major hereditary predisposing factors for AMD: the *ARMS2* [127, 128] and the *HTRA1* genes [129, 130]. At present, ARMS2 locus is considered a noncomplement-related gene because its potential role in the inflammatory process, if any, remains to be clarified [49]. In fact, although rs10490924-ARMS2 mRNA is detected in the human retina, both prevalent expression and cellular location of its putative protein are still under debate, having been initially observed in the mitochondrial outer membrane [131], and later in the cytosol and extracellular compartment [132, 133]. In any case, it seems extremely unlikely that deficiency of ARMS2-related protein could be a direct pathogenic mechanism responsible for AMD [134]. Also HTRA1 locus, encoding for a secreted protein belonging to the high-temperature requirement A family of serine proteases, can be still labeled as noncomplement-related gene [49]. However, because some molecules involved in the complement activities (i.e., clusterin, vitronectin, and fibromodulin) represent specific substrates for HTRA1 serine protease, an implication of *HTRA1* in complement system has been notionally indicated [135]. The initial investigations documented the correlation between the rs11200638 promoter variant of the HTRA1 gene and an increased expression of its protein [129, 130], whereas other studies have not replicated these outcomes [131, 136]. Nevertheless, more recent reports showed that HTRA1 mRNA expression is higher in cultured RPE cells homozygous for the HTRA1 allele related to AMD risk, also supporting the perception that *HTRA1* could be one of the causal genes in AMD patients [135, 137, 138].

In consideration of the heterogeneous gene-gene relationships between the major risk variants of the CFH, ARMS2, and HTRA1 loci, several Authors have emphasized the consistent possibility of an independent multiplicative joint effect in AMD, also taking into account that each of them should be contextualized within gene-environment interactions and epigenetic aspects [22–25, 49, 139, 140]. Exclusively limiting the focus on those well-recognized SNPs which confer increased or decreased risk of inflammation (i.e., CFH, CX3CR1, IL-8, and TLR3 and 4), and voluntarily ignoring the other, suspected or ascertained, AMD-related gene variants (i.e., APOE, ABCR, LIPC, TIMP3, PON1, ERCC6, ELOVL4, fibulin-5, hemicentin-1, SERPING1, VLDLR, LRP6, VEGF, and KDR), the etiopathogenetic scenario of AMD is exactly that of a complex/polygenic disease characterized by (i) multiple clinical phenotypes with non-Mendelian transmission; (ii) environmental effects; (iii) increased incidence with age; (iv) specific susceptibility genes with variant alleles (Table 1) [20, 22, 25, 26, 29, 46, 140–162]. The next section of this paper focuses on those immunological/inflammatory topics more directly translatable to improve the therapeutic strategies against AMD and, in particular, against its neovascular form, often responsible for the cases of most severe visual loss.

## 4. Agents Directed against the Immune Response and Age-Related Macular Degeneration

The responses of human immune system are necessary to defend our organism against several diseases, external antigens, invading microorganisms and/or acute tissue injuries. However, the contribution of the immune system in the occurrence of chronic age-related pathologic conditions has not been yet fully understood. In the course of the normal aging, as well as during chronic diseases, low-grade tissue stress (caused by noninfectious insults) may be related to subclinical damages resulting in the release of endogenous molecules, collectively called “alarmins”, that activate immunocompetent cells capable to support both innate and acquired immunity. In fact, they recruit and/or trigger receptor-expressing cells of the innate immune system, such as dendritic cells and macrophages, and consequently can also promote adaptive immunity in either direct or indirect manner [47]. To restore tissue homeostasis, by means of a mounting localized para-inflammatory response, immune system must be able to early identify such minimal bio-pathological changes in each specific district. Conversely, dysregulation or dysfunction of the immune system in chronically facing low-grade stress conditions may lead to manifest pathologies.

The retina, like the brain, is a high-metabolism tissue and is sensitive to noxious microenvironmental stimulations; however, unlike the brain, it is constantly exposed to the light, which can produce loads of photooxidized materials. Light-related stress and other oxidative damages increase in retinal tissue with the aging, as does the para-inflammatory response. In this view, although AMD is not a classic inflammatory disease, innate immunity and autoimmune components (i.e., complement factors, chemokines, cytokines, macrophages, and ocular microglia) have a reliable role in both pathogenesis and progression of AMD [47, 49, 163]. During the last two decades, directly or indirectly targeting these specific molecules/components, implicated in the immunoinflammatory pathways, has been assessed in the attempt to improve the therapeutic management of patients affected by the different clinical forms of AMD (Figures 2–4).

Since the mid 2000s, the Age-Related Eye Disease Study (AREDS, a large multicenter randomized clinical trial evaluating the long-term effects of high-dose antioxidant nutritional supplements on the incidence and progression of AMD and cataract) has documented a significantly lower incidence of advanced AMD in patients with drusen maculopathy treated with appropriate dosages of antioxidants than in a placebo group [164–169]. Also other studies and evidences indicate the opportunity to indirectly counteract para-inflammatory changes minimizing the retinal oxidative stresses in AMD patients [11, 13–15, 170, 171]. In particular, oral lutein intake results in beneficial effects on various visual function tests, and recent findings show that it is able to influence immune/inflammatory responses, not only diminishing the manifestation of various ocular inflammation models, but also suppressing NF kappa-B activation and/or inhibiting the expression of iNOS and COX-2 [48, 171].

On the other hand, during the last few years, numerous trials have been started to verify the therapeutic effects of various drugs aimed to directly downgrade the retinochoroidal immune response in AMD patients. In the next future, the outcomes of these ongoing clinical studies (156 studies found at http://clinicaltrials.gov/ searching, on June, 26 2012, with the keywords “age-related macular degeneration” and “anti-inflammatory”) together with the already reported findings [163, 172–175] will be able to provide a more exact delineation of the role of the agents directed against the immune response in therapeutic recommendations for AMD patients [165–168, 176–186]. The majority of these interventional trials are conducted on patients affected by neovascular AMD, employing corticosteroids (i.e., dexamethasone and triamcinolone acetonide), nonsteroidal anti-inflammatory drugs (i.e., low-dose acetylsalicylic acid, bromfenac, diclofenac, and nepafenac), immunosuppressive agents (i.e., methotrexate and rapamycin), and biologics (i.e., anti-TNF-*α* agents such as infliximab and adalimumab, IL-2-receptor antagonists such as daclizumab, and complement inhibitors/regulators such as ARC1905, TNX-234, eculizumab, and POT-4), with the exception of rapamycin which has been also evaluated in cases of geographic atrophy secondary to AMD [163, 187].

The pathogenetic scenario that gives rise to the first RPE-BM-CC alterations in AMD is extremely complex. It includes a variety of predisposing genetic backgrounds, which can take effect on an heterogeneous plethora of para-inflammatory causative factors: cigarette smoking, phototoxic oxidative exposure, dietary habits, alterations of iron and lipid homeostasis, buildup of advanced glycation endproducts, microbial infection, lipofuscin and beta-amyloid toxicity, excessive immune-complex generation, choroidal hemodynamic insufficiency and ischemia, phagocytic overload, and/or RPE autophagy [49]. However, regardless of what are the conditions that can initially trigger the macular degenerative pattern in each individual AMD patient, it is indisputable that the decisive downstream consequences are the deposition and/or sequestration of both cellular and acellular debris at sub-RPE level. In the course of the normal human aging, mid- or long-term malfunctions in the tissue processing of these debris can be sufficient to locally generate abnormal para-inflammatory signal with a consequent aberrant activation of the complement system. This macular status would most likely result in persistent complement attack, further sub-RPE deposits, continuous formation of drusen, bystander injury to neighboring cells and, finally, irreversible photoreceptor degeneration and/or deconstruction (especially in those lots of adult or elder individuals who are more genetically susceptible to AMD for the presence of polymorphisms influencing the immune-inflammatory pathways and, in particular, the alternative complement-modulating activity) [25, 47, 49]. Starting from this rationale, our current knowledge regarding the role of both inflammation and complement systems in AMD should be refined to the point where it can be more easily translated in an innovative enhancement of AMD treatments, by means of either comparative randomized clinical trials or interventional pilot studies or biogenetic therapeutic researches.

## 5. Final Remarks

In the recent years, a substantial amount of evidences and/or arguments document the crucial responsibility of immune-inflammatory processes in the pathogenesis of AMD [3, 47–52, 71, 188], clearly indicating the importance not only of specific complement-modulation agents, but also of nonspecific anti-inflammatory drugs, as adjunctive therapies for both non neovascular AMD (conventionally treated with AREDS formula and lutein) [163, 165–171] and, most of all, neovascular AMD (routinely treated with intravitreal administration of drugs acting against vascular endothelial growth factor (anti-VEGF) and/or with photodynamic therapy with verteporfin (PDT-V)) [163, 172–186, 189–194]. The fact that para-inflammatory dysregulation is already present in the early stage of AMD may notionally support the preventive employment of agents directed against the immune-inflammatory response in combination with high-dose nutritional supplements (particularly in those patients with a disabling form of maculopathy in one eye, younger than 65 years, and/or carrier of significant genetic susceptibility to AMD) [30, 164–171]. On the other hand, the existence of variable mid-term responsiveness of CNV to either anti-VEGF or PDT-V regimen (often resulting in elevated risks of legal blindness, high societal costs and expensive economic burden) practically recommends, above all in patients with advanced AMD in one eye, the adjunctive utilization of drugs directed against the immune-inflammatory response in combination with anti-VEGF injections and/or PDT-V [114, 171, 176–179, 183–185, 195–217].

Returning to focus on the above-mentioned translational concepts about the opportunity of pharmacologic modulation toward the immune-inflammatory pathways in AMD, a comprehensive approach is warranted to verify the chances of a prompt application of this curative modality in the clinical setting. In theory, to modulate the complement attack and minimize the local parainflammation in AMD patients who carry one or more complement-related gene polymorphisms predisposing to the disease, the most specific approach would augment the retinochoroidal bioavailability of the native/protective form of those complement factors or components responsible for the genetic susceptibility to AMD [218–220]. Adhering to this work hypothesis, a variety of delivery systems (i.e., gene transfer, cell-based therapies, organ (liver) transplantation, systemic or intraocular injections) can be envisioned to slow or arrest AMD by reasserting control over the complement system and, in particular, over its alternative pathway. If this biogenetic “augmentation” concept will be applicable also in the clinical AMD patterns, new complement-modulation therapeutics could be added to those several drugs directed against the immune-inflammatory response and already being tested on humans [49, 163]. However, at the moment, pending the concrete applicative possibilities of these biogenetic and/or pharmacologic complement-targeted treatments, open-label clinical trials are recommend, especially in patients with neovascular AMD, to better evaluate the therapeutic anti-CNV rationale in combining intravitreal corticosteroids either with the conventional anti-VEGF regimens or with anti-VEGF plus PDT-V customized protocols. In this view of good postmarketing study practice, as additional anti-CNV treatment, a promising anti-inflammatory strategy is that which involves the use of drug delivery systems (i.e., nonbiodegradable insert or biodegradable implant), able to provide a sustained release of intravitreal corticosteroids (fluocinolone acetonide or dexamethasone) for several months [221, 222]. In fact, taking into account both that immunoinflammatory phenomena are very active during the occurrence of an AMD-related CNV [71, 223], and that corticosteroids act upstream in immunoinflammatory cascades with consequent inhibition of the alternative-amplification of complement pathway [224, 225], and that a prolonged pharmacologic action represents an important parameter for the final efficacy of any therapy against neovascular AMD [176–180], the above-described intraocular devices, already approved for the treatment of peculiar forms of macular edema and of noninfectious posterior uveitis [221, 222], could represent a rational adjunctive therapeutic approach for patients with neovascular AMD undergoing repeated intravitreal injections of anti-VEGF drug.

## Figures and Tables

**Table 1:** Main AMD-susceptibility genetic loci.

Locus	Role in immunoinflammatory pathways	Possibility of AMD-risk elevation
CFH	Yes (complement system)	High (in carriers of polymorphic allele)
ARMS2	Not clarified	High (in carriers of polymorphic allele)
HTRA1	Possible (complement system)	High (in carriers of polymorphic allele)
CFB	Yes (complement system)	Intermediate (in carriers of wild allele)
C2	Yes (complement system)	Intermediate (in carriers of wild allele)
C3	Yes (complement system)	Intermediate (in carriers of polymorphic allele)
CFI	Yes (complement system)	Low (in carriers of polymorphic allele)
TIMP3	Yes (immunity in extracellular matrix)	Low (in carriers of polymorphic allele)
LIPC	Not clarified	Low (in carriers of polymorphic allele)
ABCR	No	Low (in carriers of polymorphic allele)
APOE	No	Low (in carriers of polymorphic allele)

Legend: CFH: complement factor H; ARMS2: age-related maculopathy susceptibility 2; HTRA1: high-temperature requirement factor A of serine peptidase 1; CFB: complement factor B; C2: complement component 2; C3: complement component 3; CFI: complement factor I; TIMP3: tissue inhibitor of metalloproteinases 3; LIPC: hepatic lipase gene; ABCR: ATP-binding cassette transporter; APOE: apolipoprotein E.
